# The effectiveness of community-based coordinating interventions in dementia care: a meta-analysis and subgroup analysis of intervention components

**DOI:** 10.1186/s12913-017-2677-2

**Published:** 2017-11-13

**Authors:** Amy Backhouse, Obioha C. Ukoumunne, David A. Richards, Rose McCabe, Ross Watkins, Chris Dickens

**Affiliations:** 1 University of Exeter Medical School, College House, St Luke’s Campus, Heavitree Road, Exeter, EX1 2LU UK; 2 National Institute for Health Research (NIHR) South West Peninsula Collaboration for Leadership in Applied Health Research and Care (CLAHRC), Exeter, Devon UK

**Keywords:** Dementia, Health services, Dementia care coordination, Case management, Systematic review, Meta-analysis, Collaborative care, Community interventions

## Abstract

**Background:**

Interventions aiming to coordinate services for the community-based dementia population vary in components, organisation and implementation. In this review we aimed to evaluate the effectiveness of community-based care coordinating interventions on health outcomes and investigate whether specific components of interventions influence their effects.

**Methods:**

We searched four databases from inception to April 2017: Medline, The Cochrane Library, EMBASE and PsycINFO. This was aided by a search of four grey literature databases, and backward and forward citation tracking of included papers. Title and abstract screening was followed by a full text screen by two independent reviewers, and quality was assessed using the CASP appraisal tool. We then conducted meta-analyses and subgroup analyses.

**Results:**

A total of 14 randomised controlled trials (RCTs) involving 10,372 participants were included in the review. Altogether we carried out 12 meta-analyses and 19 subgroup analyses. Meta-analyses found coordinating interventions showed a statistically significant improvement in both patient behaviour measured using the Neuropsychiatric Inventory (NPI) (mean difference (MD) = −9.5; 95% confidence interval (CI): −18.1 to −1.0; *p* = 0.03; number of studies (n) = 4; I^2^ = 88%) and caregiver burden (standardised mean difference (SMD) = −0.54; 95% CI: -1.01 to −0.07; *p* = 0.02; *n* = 5, I^2^ = 92%) compared to the control group. Subgroup analyses found interventions using a case manager with a nursing background showed a greater positive effect on caregiver quality of life than those that used case managers from other professional backgrounds (SMD = 0.94 versus 0.03, respectively; *p* < 0.001). Interventions that did not provide supervision for the case managers showed greater effectiveness for reducing the percentage of patients that are institutionalised compared to those that provided supervision (odds ratio (OR) = 0.27 versus 0.96 respectively; *p* = 0.02). There was little evidence of effects on other outcomes, or that other intervention components modify the intervention effects.

**Conclusion:**

Results show that coordinating interventions in dementia care has a positive impact on some outcomes, namely patient behaviour and caregiver burden, but the evidence is inconsistent and results were not strong enough to draw definitive conclusions on general effectiveness. With the rising prevalence of dementia, effective complex interventions will be necessary to provide high quality and effective care for patients, and facilitate collaboration of health, social and third sector services.

**Electronic supplementary material:**

The online version of this article (10.1186/s12913-017-2677-2) contains supplementary material, which is available to authorized users.

## Background

An estimated 850,000 people are living with dementia. The economic cost of dementia is estimated to be £26.3 billion in the UK alone, which is set to rise as the number of people with dementia increases [1]. In a report on dementia services in England, the National Audit Office stated that dementia had not been a public health priority, which had led to inadequate care services, poor value for money and suboptimal quality of care [2]. Furthermore, a report by the Department of Health describes a fragmentation between community services, and a lack of coordination between health and social care. The release of the National Dementia Strategy 2020 [3] was the initial step in addressing the challenges facing health and social care in improving the lives of people living with dementia.

New approaches in dementia health care have been developed to facilitate coordination, collaboration and communication in care. Strategies include assigning a case manager, usually a health or social care professional, who becomes responsible for organising and facilitating care. Such coordinating interventions improve patient outcomes in other conditions such as depression [4, 5], diabetes and coronary heart disease [6]. Similar interventions among people with dementia have provided less consistent effects. For example, some studies found coordinating interventions reduce institutionalisation for community-dwelling individuals with dementia [7, 8], whereas others have not [9, 10]. Care coordinating interventions may reduce caregiver burden and caregiver depression and improve caregiver well-being, but these effects have not been consistent and have varied across follow-up times [7]. Clinical and methodological heterogeneity across studies (subjects studied, intervention design, follow-up duration etc.) have contributed to this inconsistency, and as a result it remains unclear whether coordinating interventions can improve outcomes or what components of interventions are important.

To investigate characteristics and components of coordinating interventions for people with dementia that might improve patient and career outcomes, we recently completed a systematic review of qualitative studies [11] which investigated the views and experiences of stakeholders involved in such interventions. We identified five independent studies that encompassed the views of over 100 stakeholders including individuals with dementia, informal caregivers, general practitioners (GPs), case managers and old age psychiatrists. We identified five overarching themes associated with effective care; (1) case manager: preferences for the case manager personal and professional attributes, including a sound knowledge in dementia and availability of local services; (2) communication: the importance stakeholders placed on multichannel communication with service users, multidisciplinary teams and organisations; (3) intervention: focused primarily on the contact type and frequency between case managers and service users, and the importance of case manager training and service evaluation; (4) resources: outlined stakeholder views on the required resources for coordinating interventions and potential overlap with existing resource; and (5) support: reflected the importance that was placed on the support network around the case manager and the investment of professionals involved directly in care as well as the wider professional network.

We have conducted a systematic review and meta-analysis to evaluate the effectiveness of community-based care coordinating interventions on health outcomes of individuals with dementia and their informal caregivers. Furthermore, we investigated whether there is any evidence that potentially key components of the interventions, identified by stakeholder in studies included in our review of qualitative evidence [11], modify their effects on health outcomes of people with dementia and their carers.

## Methods

### Protocol and registration

The review protocol was registered with PROSPERO (registration: CRD42015024618), and published in BioMed Central Systematic Reviews [12] in accordance with the criteria in the Preferred Reporting Items for Systematic Reviews and Meta-Analyses (PRISMA) statement for systematic reviews [13].

### Eligibility criteria

The following criteria outline the eligibility of studies that were included in the review.

#### Types of studies

Studies were eligible if they were RCTs of community-based interventions coordinating care in dementia. We excluded non-randomised experimental studies such as before-and-after or quasi-experimental studies.

#### Types of participant

We included studies that involved participants with a dementia diagnosis of any type who were living at home, with no restrictions on age or gender. We excluded studies of individuals who did not have a formal diagnosis of dementia or had self-defined as having dementia due to the uncertainty of diagnosis in such participants. Additionally, we excluded studies that focused solely on informal caregivers of individuals with dementia which did not include a focus on increased care coordination or improved outcomes for individuals with dementia.

#### Types of intervention

We included interventions that were delivered by a single, identified professional who took responsibility for the provision and management of care. The main focus of their role was described in the study report as planning, facilitating and/or coordinating care through assessments and proactive follow-ups.

#### Control

Comparators included ‘usual care’, standard community treatment, alternative dementia care interventions or waiting-list controls.

#### Setting

We included studies of interventions that were based in the community. We excluded studies based in hospitals or nursing/residential homes, and those that involved changes made to healthcare systems or application of guidelines alone.

#### Types of outcome measures

We considered all available binary and continuous outcome measures related to individuals with dementia and/or their informal caregiver.

#### Date, language and location

No restrictions were placed on date, language or study location.

### Information sources

#### Electronic searches

The following four electronic databases were searched from date of inception to June 2015, with the search syntax being modified appropriately for the individual database: MEDLINE (OvidSP), The Cochrane Library, EMBASE and PsycINFO. Electronic searches were updated in April 2017.

#### Additional resources

We searched four additional databases for unpublished studies; the Health Management Information Consortium (HMIC), Social Policy and Practice (SPP), ProQuest and the International Clinical Trials Registry Platform (ICTRP). Backward and forward citation searches were completed on included studies and relevant systematic reviews identified in screening.

### Search

A comprehensive search strategy was developed through consultation with an information specialist (DM) and information on intervention terminology from a prior scoping review of the literature. The search based on the outlined eligibility criteria used a combination of controlled vocabulary specific to the individual database (e.g. MEDLINE Medical Subject Headings (MeSH terms)) and free text terms. A master search strategy can be found in Additional file 1.

### Study selection

#### Data management

All references were managed in EndNote X7.0.2. Titles and abstracts of studies identified in the initial search were imported into EndNote and duplicates were removed, then full texts of potentially relevant papers were imported for further screening.

#### Screening

Two independent reviewers (AB, RW) conducted an initial screening of titles and abstracts followed by a screening of potential relevant full texts guided by inclusion criteria. A third reviewer (CD) was available for any screening disagreement.

### Data extraction

A bespoke data extraction sheet designed using Microsoft Office Excel was piloted by one reviewer (AB) on three RCTs and modified in light of piloting. Data were extracted on study design, participant characteristics, methodology, intervention characteristics, comparator group(s) and outcome measures.

Data were also extracted on intervention components identified as potentially important in influencing treatment effects, based on the results of our recent review of qualitative evidence [11]. Informed by our review of qualitative studies the following intervention characteristics were identified for subgroup analysis:

1. Case manager base – i.e. the working location of the case manager, either in community or non-community (e.g. primary care) settings
2. Case manager professional background – nursing background or non-nursing
3. Case manager training – specifically trained for the case manager role or not
4. Contact frequency – how often the case manager was in contact, grouped as those with low contact frequency (less than or equal to the median across studies of 14.4 contacts per 12 months) and high contact frequency (more than 14.4 contacts per 12 months).
5. Contact type – mode of contact (i.e. telephone, face-to-face or written) used to communicate with individuals with dementia and their caregivers
6. Supervision – whether or not the case manager had been assigned a mentor or supervisor during the intervention
7. Workload – case manager caseload, divided at mean number of patients per case manager across studies (51.1), into those with high caseload (more than 50 patients) and low caseload (less than or equal to 50 patients).

Results of studies represented in multiple papers are included in the review once to avoid double counting. For trials with more than one associated paper, the primary paper has been cited as the main reference though data were extracted from all available papers. We approached authors via email to obtain missing data. Six missing standard deviations (SD) were calculated from standard errors of the mean (SEM) and two missing SDs were obtained from other studies.

### Risk of bias

The Critical Appraisal Skills Programme (CASP) RCT appraisal tool [14] was used to assess the quality of included studies. The checklist includes 11 questions covering rigour, research methods, relevance and research integrity. Two independent reviewers (AB, RW) assessed the quality of included trials, and disagreement was resolved through discussion.

### Method of analysis

Descriptive statistics were used to summarise main study characteristics and the risk of bias.

#### Meta-analysis

Random-effects meta-analyses of RCTs were conducted using Review Manager 5.3. Random-effects meta-analysis was selected over fixed-effect meta-analysis because of the methodological heterogeneity across studies. For continuous outcomes, standardised mean differences (SMD) were pooled, except when change scores and final scores were combined in which case the mean difference (MD) was pooled. For binary outcomes, odds ratios (OR) were pooled. For the purpose of this meta-analysis, where specific outcomes were measured across multiple time points, the result nearest the median time point for that outcome was used. Heterogeneity across studies was quantified using the I squared (I [2]) statistic (the percentage of variation across studies that is due to between-study heterogeneity as opposed to chance) [15].

#### Subgroup analysis

Trials were grouped based on the presence or absence of intervention components identified, as outlined above. Intervention effects were estimated within subgroups and compared across subgroups to identify components for which the size of the effect depends on whether they are present; in other words, to identify components that modify the intervention effects.

## Results

### Study selection

**Fig. 1:**
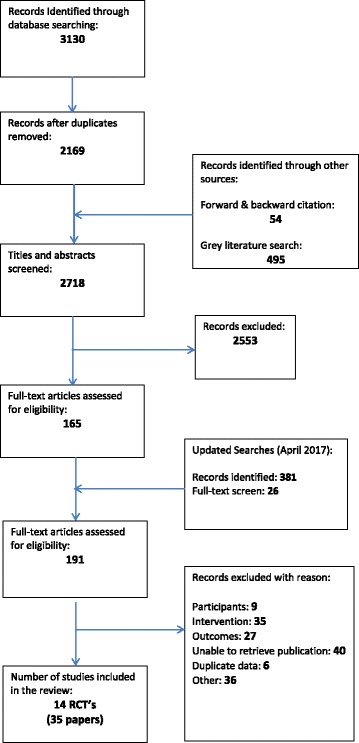
Study flow diagram (PRISMA)

The original search identified 2718 citations, and an updated search performed in April 2017 identified a further 381 records for screening. 191 citations underwent a full-text screen by two independent reviewers (AB, RW), and a total of 35 papers from 14 trials were included in the final review (see Additional file 2). A full report of the selection process can be found in the PRISMA diagram in Fig. 1.

### Study characteristics

Of the 14 trials, six were based in the USA [16–21], three in China [22–24], two in Finland [25, 26], and one each in the Netherlands [27], India [28] and Canada [29]. In total, the trials included 10,372 participants, with 8095 recruited from one trial [19]. Three trials randomised clusters [17, 18, 21] and 11 [16, 19, 20, 22–27] randomised individuals. The duration of the trials ranged from 4 months to over 2 years. Trial characteristics are summarised in Table 1.

**Table 1:** Study characteristics

Study ID	Country	Randomisation unit	Sample Size		Intervention duration	Length of follow-up
			Intervention	Control		
Bass 2003	USA	Individual	94	63	12 months	12 months
Bass 2014	USA	Clinic	316	192	12 months	12 months
Callahan 2006	USA	Physician	84	69	12 months	18 months
Chien 2008	China	Individual	46	46	6 months	12 months
Chien 2011	China	Individual	44	44	6 months	18 months
Chu 2000	Canada	Individual	37	38	18 months	18 months
Dias 2008	India	Individual	41	40	6 months	6 months
Eloniemi-Sulkava 2001	Finland	Individual	53	47	2 year	2 year
Eloniemi-Sulkava 2009	Finland	Individual	63	62	2 year	2 year
Jansen 2011	The Netherlands	Individual	54	45	12 months	12 months
Lam 2009	China	Individual	59	43	4 months	12 months
Newcomer 1999	USA	Individual	4151	3944	NR	36 months
Samus 2014	USA	Individual	106	183	18 months	18 months
Vickrey 2006	USA	Clinic	238	170	4–16 months	18 months

Eight of the trials had case managers based in community teams [16, 20–24, 27, 28], two trials used case managers in both community and primary care teams [17, 19], in two trials [18, 25] case managers were based in primary care and two trials [26, 29] did not report case manager base. Six trials used a nurse case manager [18, 22, 23, 25–27], four trials used social workers [16, 20, 21, 29] and two trials [17, 19] used a combination of the two. One trial used an occupational therapist [24]. Nine trials [17, 20–23, 25–28] reported on specified training around the role for case managers, four [16, 19, 24, 29] did not report any training and only one trial [18] clarified no training. All but two of the trials [16, 17] used both face-to-face and telephone contact as forms of communication between case managers and service users, with two trials using telephone contact only [16, 17]. Of the 14 trials, seven [16–20, 26, 28] reported providing supervision to case managers, four trials [21, 24, 27, 29] did not report on supervision and three [22, 23, 25] clearly stated no supervision was provided. Further details of trial components can be found in Additional file 3.

### Risk of bias

All of the trials were rated as high or moderate quality, and all had used appropriate methods for randomisation and were therefore free of selection bias. Results of the CASP appraisal can be found in Additional file 4.

### Analysis results

Binary outcomes included hospitalisation (whether or not the patient was admitted to hospital), institutionalisation (whether or not the patient was admitted to a residential or nursing home) and mortality. Continuous outcomes for people with dementia included quality of life, behaviour, cognition, depression and function. Continuous outcomes for caregivers included quality of life, mood, burden and social support. Forest plots for each comparison can be found in Additional file 5.

#### Meta-analysis

Coordinating interventions showed a statistically significant improvement in both patient behaviour measured using the NPI (MD = −9.5; 95% confidence interval (CI): −18.1 to −1.0; *p* = 0.03; number of studies (n) = 4; I^2^ = 88%) and caregiver burden (SMD = −0.54; 95% CI: -1.01 to −0.07; *p* = 0.02; *n* = 5, I^2^ = 92%) compared to the control group (See Table 2). There was only weak evidence of effects on institutionalisation (OR = 0.60; 95% CI: 0.32 to 1.11; *p* = 0.10; *n* = 9; I^2^ = 48%), caregiver mood (SMD = −0.04; 95% CI; −0.10 to 0.01; p = 0.10; *n* = 6; I^2^ = 0%), caregiver quality of life (SMD = 0.45; 95% CI: -0.03 to 0.94; *p* = 0.07; *n* = 4; I^2^ = 89%) and social support (SMD = 0.38; 95% CI: -0.08 to 0.84; p = 0.10; *n* = 3; I^2^ = 81%), and little evidence of effects for hospitalisation (*p* = 0.50), mortality (*p* = 0.73), patient quality of life (*p* = 0.35), patient cognition (*p* = 0.40), patient depression (*p* = 0.48) or patient function (*p* = 0.46).

**Table 2:** Effects of coordinating interventions on binary and continuous outcome measures

Outcome Measure	Number of trials included	Follow-up time point range	Odds Ratio	95% CI	I^2^	*P* Value
Patient Hospitalisation	6	12–18 months	0.89	0.64 to 1.25	0%	0.50
Patient Institutionalisation	9	10–12 months	0.60	0.32 to 1.11	48%	0.10
Patient Mortality	9	6–12 months	0.97	0.81 to 1.16	0%	0.73
Outcome Measure	Number of trials included		Standardised Mean Difference	95% CI	I^2^	*P* Value
Patient Quality of Life	3	12 months	0.09	−0.09 to 0.27	0%	0.35
Patient Cognition	4	12 months	−0.09	−0.29 to 0.11	0%	0.40
Patient Function	3	6 months	−0.08	−0.30 to 0.14	0%	0.46
Caregiver Burden	5	6–18 months	−0.54	−1.01 to −0.07	92%	0.02
Caregiver Mood	6	6–18 months	−0.04	−0.10 to 0.01	0%	0.10
Caregiver Quality of Life	4	9–12 months	0.45	−0.03 to 0.94	89%	0.07
Social Support	3	12 months	0.38	−0.08 to 0.84	81%	0.10
Outcome Measure	Number of trials included		Mean Difference	95% CI	I^2^	*P* Value
Patient Behaviour	4	12 months	−9.52	−18.05 to −1.00	88%	0.03
Patient Depression	3	9–12 months	0.60	−1.08 to 2.27	66%	0.48

*CI* Confidence intervals, *I* ^*2*^ I squared statistic, *MD* Mean difference, *SMD* Standardised mean difference

#### Subgroup analysis

Interventions using a case manager with a nursing background showed a greater positive effect on caregiver quality of life compared to those that used other professional backgrounds (SMD = 0.94 versus 0.03, respectively; *p* < 0.001). Interventions that did not provide case managers with supervision showed greater effectiveness for reducing the percentage of patients that are institutionalised compared to those that provided supervision (OR = 0.27 versus 0.96 respectively; *p* = 0.02). There was weak evidence that interventions using a lower caseload for case managers had greater effectiveness for reducing the number of patients institutionalised compared to interventions using a higher caseload for case managers (OR = 0.23 versus 1.20 respectively; *p* = 0.08). There was little evidence that the other intervention components modify treatment effects (see Table 3).

**Table 3:** Subgroup analysis of intervention components

Outcome Measure	Subgroups	Number of Trials Included	Odds Ratio	95% CI	I^2^	*p* Value*
Hospitalisation	Community base	2	1.21	0.70 to 2.08	0%	
	Non-community base	4	0.74	0.48 to 1.13	0%	
	Subgroup difference					0.16
	Nursing background	4	0.96	0.58 to 1.60	9%	
	Non-nursing background	2	0.81	0.51 to 1.31	0%	
	Subgroup difference					0.63
	High contact	2	0.49	0.19 to 1.30	0%	
	Low contact	3	0.97	0.61 to 1.53	19%	
	Subgroup difference					0.22
	Supervision	3	0.99	0.60 to 1.62	19%	
	No supervision	3	0.78	0.47 to 1.30	0%	
	Subgroup difference					0.52
	Low workload	4	0.74	0.48 to 1.13	0%	
	High workload	2	1.21	0.70 to 2.08	0%	
	Subgroup difference					0.16
Institutionalisation	Community base	4	0.50	0.16 to 1.61	37%	
	Non-community base	2	0.93	0.09 to 9.06	70%	
	Subgroup difference					0.64
	Nursing background	6	0.44	0.20 to 0.95	23%	
	Non-nursing background	2	0.69	0.10 to 4.98	49%	
	Subgroup difference					0.67
	High contact	3	0.38	0.11 to 1.34	39%	
	Low contact	3	1.05	0.21 to 5.18	43%	
	Subgroup difference					0.33
	Supervision	3	0.96	0.47 to 1.95	26%	
	No supervision	3	0.27	0.12 to 0.61	0%	
	Subgroup difference					0.02
	Low workload	2	0.23	0.08 to 0.67	0%	
	High workload	3	1.20	0.27 to 5.32	37%	
	Subgroup difference					0.08
Mortality	Community base	4	0.74	0.36 to 1.51	0%	
	Non-community base	2	1.71	0.55 to 5.30	0%	
	Subgroup difference					0.22
	Nursing background	4	1.44	0.65 to 3.15	0%	
	Non-nursing background	3	1.42	0.48 to 4.22	0%	
	Subgroup difference					0.99
	High contact	2	0.62	0.23 to 1.65	25%	
	Low contact	4	1.50	0.55 to 4.10	0%	
	Subgroup difference					0.22
	Low workload	2	0.50	0.18 to 1.36	0%	
	High workload	3	1.26	0.52 to 3.03	0%	
	Subgroup difference					0.17
Outcome Measure	Subgroups	Number of Trials Included	SMD	95% CI	I^2^	*p* Value*
Patient Cognition	Community base	2	0.06	−0.23 to 0.35	0%	
	Non-community base	2	−0.21	−0.49 to 0.06	0%	
	Subgroup difference					0.18
Caregiver Burden	Supervision	2	−0.78	−1.69 to 0.14	96%	
	No Supervision	2	−0.24	−0.49 to 0.02	0%	
	Subgroup difference					0.27
Caregiver Mood	High contact	2	−0.05	−0.11 to 0.00	0%	
	Low contact	4	0.01	−0.13 to 0.16	1%	
	Subgroup difference					0.41
Caregiver quality of life	Nursing background	2	0.94	0.63 to 1.25	0%	
	Non-nursing background	2	0.03	−0.32 to 0.39	89%	
	Subgroup difference					< 0.001
Outcome Measure	Subgroups	Number of Trials Included	MD	95% CI	I^2^	*p* Value*
Patient Behaviour	Low workload	2	−13.2	−28.23 to 1.75	95%	
	High workload	2	−5.4	−10.63 to −0.17	0%	
	Subgroup difference					0.33

*CI* Confidence intervals, *I* ^*2*^ I squared statistic, *MD* Mean difference*, SMD Standardised mean*

*p value is for the subgroup comparisons

#### Publication bias

Publication bias was explored using funnel plots (Additional file 6). Institutionalisation and mortality were the only two outcome measures to show a positive-result publication bias, the results of neither were statistically significant in the meta-analysis of overall intervention effect.

## Discussion

### Summary of evidence

In this review we conducted a meta-analysis of binary and continuous outcomes reported in 14 RCTs to explore the effectiveness of coordinating interventions in dementia care. The results from the meta-analyses demonstrated that coordination interventions have a varying degree of effect on a variety of outcomes. The effects of coordinating interventions appear to be a reduction in caregiver burden and improvements in patient behaviours.

Of the intervention components that were analysed, case manager professional background and supervision were the only ones for which there was evidence that they modify the intervention effect. The difference in effect sizes found in the analysis are considered large, and therefore likely to be clinically significant based on the criteria set out by Cohen [30]. Lack of an identified supervisor for case managers is associated with a greater reduction in institutionalisation rates and case managers with a nursing background, as opposed to other professional backgrounds such a social work or occupational therapy, are associated with improved caregiver quality of life.

Our meta-analysis findings are consistent with previous reviews [6–9] of coordinating interventions in dementia care in painting a varying and complex view of the effects these interventions have on patient and caregiver outcomes. Our subgroup analyses are consistent with findings of Bower et al. 2006 [31], who reported that in trials of collaborative care for depression specific mental health background of case managers predicted improvements in depressive symptoms. However, the finding that no supervision was associated with a greater reduction in institutionalisation was inconsistent with Bower’s [31] finding that providing supervision also predicted improvements in depressive symptoms. It is possible that the line management that was provided to individuals taking on the case manager role in trials was a sufficient supportive structure for the role. However, supervision is an important structure and standard practice not just in coordinating interventions but also in many clinical roles.

Although previous research has highlighted that stakeholders have preferences in the structure, delivery and components of coordinating interventions, there is little evidence to support the notion that incorporating the preferences will have a positive impact on patient and caregiver outcomes. However, it is possible this is a function of the trial design, and that in the included trials they had not intentionally set out to include stakeholder preferences.

### Limitations

Although authors were contacted for missing information that was not included in the text, including on intervention components, there was substantial variability in the outcome measures recorded, the interventions and the reporting of the necessary intervention components which meant that only a small number of trials could be included in many of the meta-analyses and subgroup analyses. As a result, the confidence intervals for the intervention effect are often wide indicating that no effect or at the other extreme a larger effect are both plausible truths for some outcomes.

Although trials were grouped and their effects compared based on whether they included a specific component, the nature of a given component differed across trials. This variability within intervention components across trials needs to be considered in the interpretation of the meta-analyses results.

The quality of the included trials varied but the majority used appropriate methods for randomisation and were therefore free of selection bias. However, due to the nature and complexity of coordinating interventions, most of the participants and professionals involved in the interventions were not blinded leading to potential detection bias. This issue was reduced in the majority of trials through the use of self-report measures, service use data or a blinded external data collector. There was an indication of potential publication bias for two outcome measures, suggesting there is a possibility that smaller trials that found negative results were not published.

### Future research

From the results of this meta-analysis, and existing systematic reviews [6–9], evidence for coordinating interventions in dementia appears inconsistent. There is potential room to address the differences in coordinating intervention models in order to clarify and synchronise their aims, structure and implementation. However, an important message from this review is the importance of future trials of any complex intervention to be rigorous in their design and implementation, and focus on high quality reporting not only of research methods but of the intervention details. It is important that the content of the intervention is comprehensively described to allow replication and comparison across trials.

The results from subgroup analyses could have interesting implications for future design of coordinating interventions. Using case managers with a nursing background and assigning a low caseload, such as a maximum of 50 patients per case manager, in new coordinating interventions could be beneficial for implementation and outcomes. Institutionalisation showed a statistically significant effect in one subgroup analysis, therefore incorporating this in the overarching aims of coordinating interventions and implementing components with a focus on delaying institutionalisation could help improve the success of intervention trials.

## Conclusions

The results of our review have shown that coordinating interventions have some potential for positive impact on selected outcome measures, but the evidence is inconsistent. The differences across models of coordinating interventions in dementia care are substantial, and this has made it difficult to identify what should be considered core components. However, with the rising prevalence of dementia, it is likely that complex interventions will be necessary to provide high quality and effective care for patients, and facilitate collaboration of health, social and third sector services. Furthermore, although there are challenges to the implementation of coordinating interventions, addressing those and incorporating more stakeholder preferences may produce more consistent results and increase the likelihood of success.

## Additional files

Additional file 1: Master search strategy – search deployed in MEDLINE OvidSP database. (DOCX 13 kb)

Additional file 2: All included papers – outlines the papers 35 papers associated to each of the 14 trials. (DOCX 20 kb)

Additional file 3: Trial components – details the components used in the subgroup analyses and how each trial would have been grouped. (XLSX 14 kb)

Additional file 4: Risk of bias – details the full response to the 11 CASP criteria assessing quality of RCT’s for each of the included trials, and details the scoring and rating of each trial. (XLSX 10 kb)

Additional file 5: Forest plots – all of the forest plots for the meta-analyses and the subgroup analyses. (DOCX 1725 kb)

Additional file 6: Publication bias – all of the funnel plots to assess publication bias for each outcome measure. (DOCX 144 kb)

## Abbreviations

CASP: Critical Appraisal Skills Programme
CI: Confidence Interval
CLAHRC: Collaboration for Leadership in Applied Health Research and Care
GP: General Practitioner(s)
MD: Mean Difference
NHS: National Health Service
NIHR: National Institute for Health Research
NPI: Neuropsychiatric Inventory
OR: Odds Ratio
RCTs: Randomised Controlled Trial(s)
SD: Standard Deviation
SEM: Standard Error of the Mean
SMD: Standardised Mean Difference
