# Supplementary material for: The effectiveness of community-based coordinating interventions in dementia care: a meta-analysis and subgroup analysis of intervention components
Source: BMC Health Serv Res. 2017 Nov 13;17:717. doi: 10.1186/s12913-017-2677-2 (PMC5683245; doi:10.1186/s12913-017-2677-2)
Supplement: Supplementary file 1 — Master search strategy – search deployed in MEDLINE OvidSP database. (DOCX 13 kb) [file 12913_2017_2677_MOESM1_ESM.docx]

**Appendix 1: Master Search Strategy in MEDLINE (OvidSP)**

Database: Ovid MEDLINE(R) In-Process & Other Non-Indexed Citations and Ovid MEDLINE(R) <1946 to Present>

Search Strategy:

--------------------------------------------------------------------------------

1 exp Dementia/

2 dement*.mp.

3 alzheimer*.mp.

4 (presenile/ or senile.mp.) and dement*.mp. [mp=title, abstract, original title, name of substance word, subject heading word, keyword heading word, protocol supplementary concept word, rare disease supplementary concept word, unique identifier]

5 *Delirium, Dementia, Amnestic, Cognitive Disorders/

6 *cognition disorders/ or *mild cognitive impairment/

7 1 or 2 or 3 or 4 or 5 or 6

8 Case Management/

9 collaborative care.mp.

10 case manag*.ti,ab.

11 care manag*.ti,ab.

12 (care adj2 coordinat*).ti,ab.

13 (case adj2 coordinat*).ti,ab.

14 service coordinat*.ti,ab.

15 care consult*.ti,ab.

16 case consult*.ti,ab.

17 (care adj2 facilitat*).ti,ab.

18 shared care.ti,ab.

19 (coordinat* adj2 care).ti,ab.

20 admiral nursing.mp.

21 *disease management/

22 8 or 9 or 10 or 11 or 12 or 13 or 14 or 15 or 16 or 17 or 18 or 19 or 20 or 21 (33785)

23 7 and 22

***************************

[mp=title, abstract, original title, name of substance word, subject heading word, keyword heading word, protocol supplementary concept word, rare disease supplementary concept word, unique identifier]

[ti,ab= title & abstract]
