# Supplementary material for: The effectiveness of community-based coordinating interventions in dementia care: a meta-analysis and subgroup analysis of intervention components
Source: BMC Health Serv Res. 2017 Nov 13;17:717. doi: 10.1186/s12913-017-2677-2 (PMC5683245; doi:10.1186/s12913-017-2677-2)
Supplement: Supplementary file 2 — All included papers – outlines the papers 35 papers associated to each of the 14 trials. (DOCX 20 kb) [file 12913_2017_2677_MOESM2_ESM.docx]

**Appendix 3: Study IDs and associated papers included in the review**

| ***Study ID*** | ***Associated papers used in the review*** |
| --- | --- |
| Bass 2003 | Bass DM, Clark PA, Looman WJ, McCarthy CA, Eckert S. The Cleveland Alzheimer’s Managed Care Demonstration: outcomes after 12 months of implementation. The Gerontologist. 2003;43(1): 73-85. |
|  | Clark PA, Bass DM, Looman WJ, McCarthy CA, Eckert S. Outcomes for patients with dementia from the Cleveland Alzheimer's managed care demonstration. Aging & Mental Health. 2004;8(1): 40-51. |
| Bass 2014 | Bass DM, Judge KS, Snow AL, Wilson NL, Morgan RO, Maslow K, Randazzo R, Moye JA, Odenheimer GL, Archambault E, Elbein R, Pirraglia P, Teasdale TA, McCarthy CA, Looman WJ, Kunik ME. A controlled trial of Partners in Dementia Care: veteran outcomes after six and twelve months. Alzheimer’s Research & Therapy. 2014;6(9): DOI: 10.1186/alzrt242 |
|  | Judge KS, Bass DM, Snow AL, Wilson NL, Morgan R, Looman WJ, McCarthy C, Kunik ME. Partners in Dementia Care: a care coordination intervention for individuals with dementia and their families. The Gerontologist. 2011;51(2): 261-272. |
|  | Shrestha S, Judge KS, Wilson NL, Moye JA, Snow AL, Kunik ME. Utilization of legal and financial services of Partners in Dementia Care study. American Journal of Alzheimer's Disease and Other Dementias. 2011;26(2): 115-120. |
|  | Bass DM, Judge KS, Snow AL, Wilson NL, Looman WJ, McCarthy C, Morgan R, Ablorh-Odjidja C, Kunik ME. Negative caregiving effects among caregivers of veterans with dementia. American Journal of Geriatric Psychiatry. 2012;20(3): 239-247. |
|  | Bass DM, Judge KS, Snow AL, Wilson NL, Morgan R, Looman WJ, McCarthy CA, Maslow K, Moye JA, Randazzo R, Garcia-Maldonado M, Elbein R, Odenheimer G, Kunik ME. Caregiver outcomes of Partners in Dementia Care: effect of a care coordination program for veterans with dementia and their family members and friends. American Journal of Geriatric Psychiatry. 2013;61: 1377-1386. |
|  | Morgan R, Bass DM, Judge KS, Liu CF, Wilson N, Snow AL, Pirraglia P, Garcia-Maldonado M, Raia P, Fouladi NN, Kunik ME. A break-even analysis for dementia care coodrination: Partners in Dementia Care. Journal of General Internal Medicine. 2015;30(6): 804-809. |
| Callahan 2006 | Callahan CM, Boustani MA, Frederick FW, Austrom GM, Damush TM, Perkins AJ, Fultz BA, Hui SL, Counsell SR, Hendrie HC. Effectiveness of collaborative care for older adults with Alzheimer’s disease in primary care: a randomised controlled trial. The Journal of the American Medical Association. 2006;295(18): 2148-2157. |
|  | Guerriero-Austrom M, Damush TM, Hartwell CW, Perkins T, Unverzagt F, Boustani M, Hendrie HC, Callahan CM. Development and implementation of nonpharmacological protocols for the management of patients with Alzheimer's disease and their families in a multiracial primary care setting. The Gerontologist. 2004;44(4): 548-553. |
| Chien 2008 | Chien WT, Lee YM. A disease management program for families of persons in Hong Kong with dementia. Psychiatric Services. 2008;59(4): 433-436. |
| Chien 2011 | Chien WT, Lee IY. Randomised controlled trial of a dementia care programme for families of home-resided older people with dementia. Journal of Advanced Nursing. 2011;67(4): 774-787. |
| Chu 2000 | Chu P, Edwards J, Levin R, Thompson J. The use of clinical case management for early stage Alzheimer’s patients and their families. The American Journal of Alzheimer’s Disease and Other Dementia. 2000;15(5): 284-290. |
| Dias 2008 | Dias A, Dewey ME, D’Souza J, Dhume R, Motghare DD, Shaji KS, Menon R, Prince M, Patel V. The effectiveness of a home care programme for supporting caregivers of persons with dementia in developing countries: a randomised controlled trial from Goa, India. PLoS One. 2008;4(6): e2333. |
| Eloniemi-Sulkava 2001 | Eloniemi-Sulkava U, Notkola IL, Hentinen M, Kivela SL, Sivenius J, Sulkava R. Effects of supporting community-living demented patients and their caregivers: a randomised trial. Journal of the American Geriatrics Society. 2001;49: 1282-1287. |
| Eloniemi-Sulkava 2009 | Eloniemi-Sulkava U, Saarenheimo M, Laakonen ML, Pietila M, Savikko N, Kautiainen H, Tilvis RS, Pitkala KH. Family care as collaboration: effectiveness of a multicomponent support program for elderly couples with dementia. Randomised controlled intervention study. Journal of the American Geriatrics Society. 2009;57: 2200-2208. |
| Jansen 2011 | Jansen APD, van Hout HPJ, Nijpels G, Rijmen F, Droes RM, Pot AM, Schellevis FG, Stalman WAB, van Marwijk HWJ. Effectiveness of case management among older dults with early symptoms of dementia and their primary informal caregivers: a randomised clinical trial. Internaitonal Journal of Nursing Studies. 2011;48: 933-943. |
| Lam 2009 | Lam CW, Lee JSW, Chung JCC, Lau A, Woo J, Kwok TCY. A randomised controlled trial to examine the effectiveness of case management model for community dwelling older persons with mild dementia in Hong Kong. International Journal of Geriatric Psychiatry. 2010;25: 395-402. |
|  | Kwok T, Lam L, Chung J. Case management to improve quality of life of older people with early dementia and to reduce caregiver durden. Hong Kong Medical Journal. 2012;18(6): s4-6. |
| Newcomer 1999 | Newcomer R, Miller R, Clay T, Fox P. Effects of the Medicare Alzheimer’s Disease Demonstration on Medicare expenditures. Health Care Financing Review. 1999;20(4): 45-65. |
|  | Yordi C, DuNah R, Bostrom A, Fox P, Wilkinson A, Newcomer R. Caregiver supports: outcomes from the Medicare Alzheimer's disease demonstration. Health Care Financing Reviw. 1997;19(2): 97-117. |
|  | Arnsberger P, Fox P, Zhang X. Case manager-defined roles in the Medicare Alzheimer's disease demonstration: relationship to client and caregiver outcomes. The Care Management Journals. 1999;1(1): 30-37. |
|  | Miller R, Newcomer R, Fox P. Effects of the Medicare Alzheimer's disease demonstration on nursing home entry. Health Service Research. 1999;34(3): 691-714. |
|  | Newcomer R, Spitalny M, Fox P, Yordi C. Effects of the Medicare Alzheimer's disease demonstration on the use of community-based services. Health Services Research. 1999;34(3): 645-667. |
|  | Newcomer R, Yordi C, DuNah R, Wilkinson A. Effects of the Medicare Alzheimer's disease demonstration on caregiver burden and depression. Health Services Research. 1999;34(3): 669-689. |
|  | Fox P, Newcomer R, Yordi C, Arnsberger P. Lessons learned from the Medicare Alzheimer disease demonstrations. Alzheimer Disease and Associated Disorders. 2000;14(2): 87-93. |
|  | Gaugler JE, Kane RL, Kane RA, Newcomer R. Early community-based service utilization and its effects on institutionalisation in dementia caregiving. The Gerontologist. 2005;45(2): 177-185. |
| Samus 2014 | Samus QM, Johnston D, Black BS, Hess E, Lyman C, Vavilikolanu A, Pollutra J, Leoutsakos JM, Gitlin LN, Rabins PV, Lyketsos CG. A multidimensional home-based care coordination intervention for elders with memory disorders: the maximising independence at home (MIND) pilot randomised trial. American Journal of Geriatric Psychiatry. 2014;22(4): 398-414. |
|  | Tanner JA, Black BS, Johnston D, Hess E, Leoutsakos JM, Gitlin LN, Rabins PV, Lyketsos CG, Samus QM. A randomiszed controlled trial of a community-based intervention: effects of MIND at home on caregiver outcomes. American Journal of Geriatric Psychiatry. 2015;23(4): 391-402. |
| Vickrey 2006 | Vickrey BG, Mittman BS, Connor KI, Pearson ML, Della Penna RD, Ganiats TG, Demonte RW, Chodosh J, Cui X, Vassar S, Duan N, Lee M. The effect of a disease management intervention on quality and outcome of dementia care: a randomised trial. Annals of Internal Medicine. 2006;145(10): 713-726. |
|  | Chodosh J, Berry E, Lee M, Connor K, DeMonte R, Ganiats T, Heikoff L, Rubenstein L, Mittlman B, Vickrey B. Effect of a dementia care management intervention on primary care provider knowledgem attitudes and perceptions of quality of care. American Journal of Geriatric Psychiatry. 2006;54(2): 311-317. |
|  | Duru KO, Ettner SL, Vassar SD, Chodosh J, Vickrey B. Cost evaluation of a coordinated care management intervention for dementia. The American Journal of Managed Care. 2009;15(8): 521-528. |
|  | Chodosh J, Pearson ML, Connor KI, Vassar SD, Kaisey M, Lee ML, Vickrey B. A dementia care management intervention: which components improve quality? The American Journal of Managed Care. 2012;18(2): 85-94. |
|  | Kaisey M, Mittman B, Pearson M, Connor KI, Chodosh J, Vassar SD, Nguyen FT, Vickrey B. Predictors of acceptance of offered care management intervention services in a quality improvement trial for dementia. International Journal of Geriatric Psychiatry. 2012;27: 1078-1085. |
|  | Brown AF, Vassar SD, Connor KI, Vickrey B. Collaborative care management reduces disparities in dementia care quality for caregiver with less education. American Journal of Geriatric Psychiatry. 2013;61: 243-251. |
