# Supplementary material for: The effectiveness of community-based coordinating interventions in dementia care: a meta-analysis and subgroup analysis of intervention components
Source: BMC Health Serv Res. 2017 Nov 13;17:717. doi: 10.1186/s12913-017-2677-2 (PMC5683245; doi:10.1186/s12913-017-2677-2)

**Appendix 1: Forest plots for meta-analyses and subgroup analyses**

**Meta-analyses comparison: Coordination intervention versus control**

**Outcome 1: Hospitalisation**


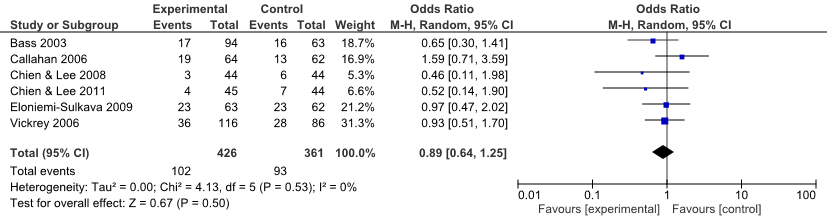


**Outcome 2: Institutionalisation**


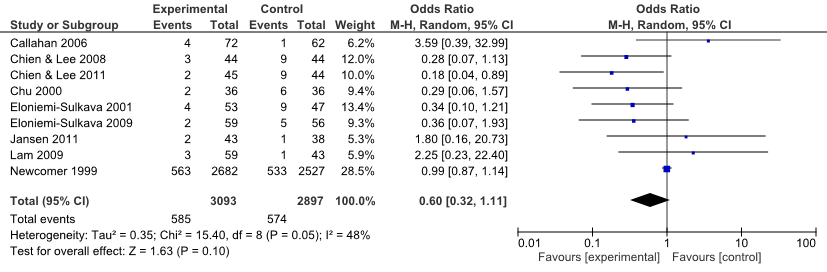


**Outcome 3: Mortality**


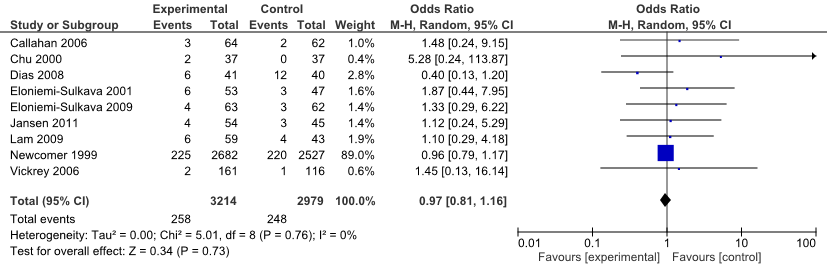


**Outcome 4: Patient quality of life**


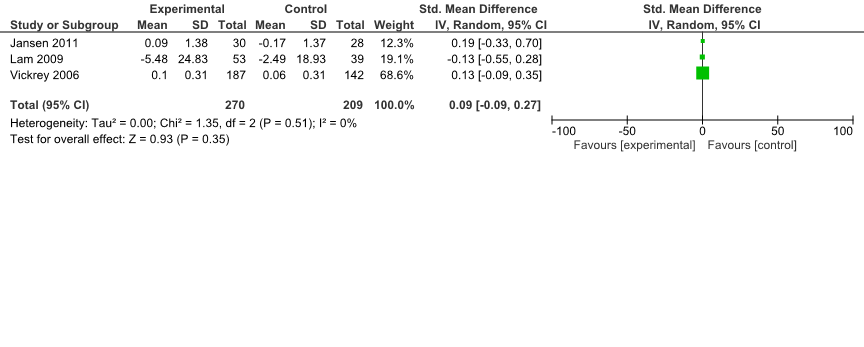
**Outcome 5: Patient cognition**


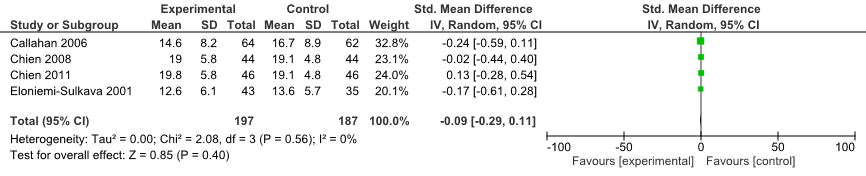


**Outcome 6: Patient function**


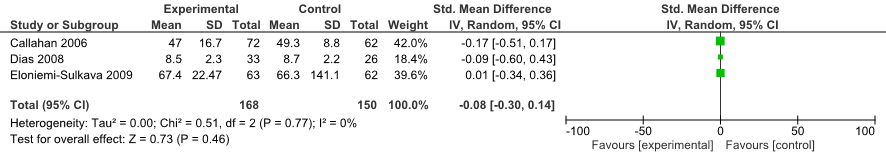


**Outcome 7: Caregiver burden**


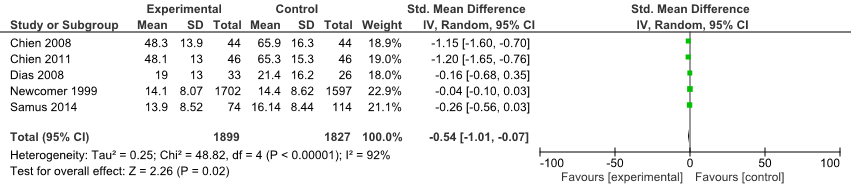


**Outcome 8: Caregiver mood**


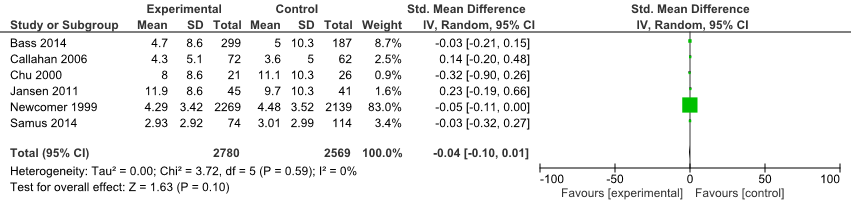


**Outcome 9: Caregiver quality of life**


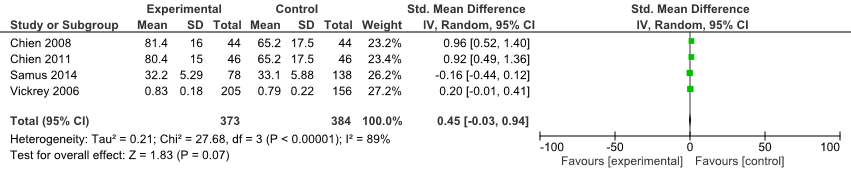


**Outcome 10: Social support**


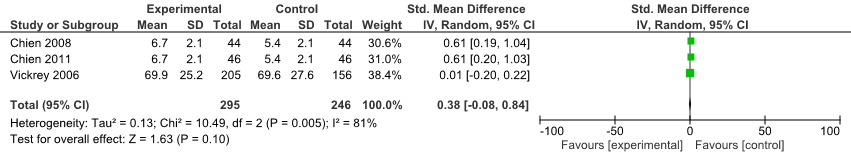


**Outcome 11: Patient behaviour**


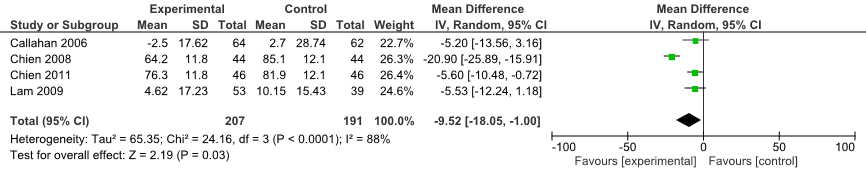


**Outcome 12: Patient depression**


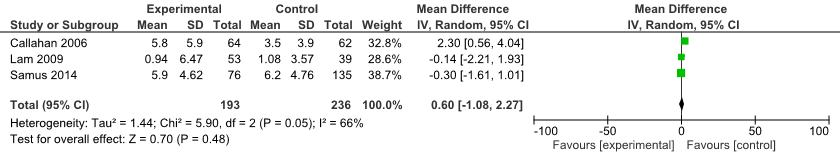


**Subgroup analyses**

**Outcome 1: Hospitalisation**

**Comparison 1: Community base versus non-community base case managers**


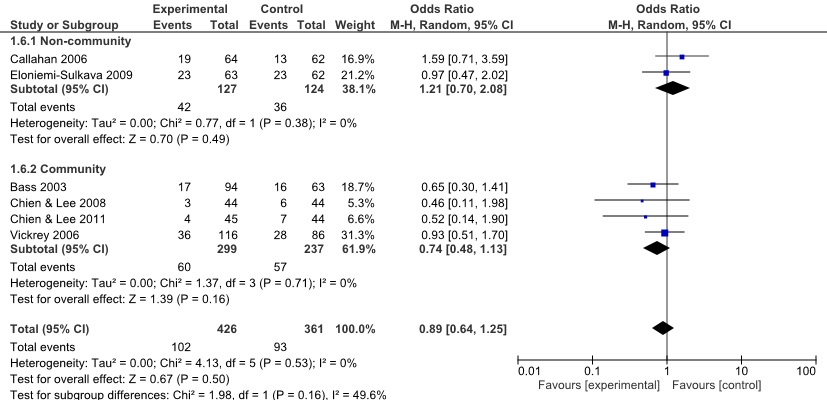


**Comparison 2: Nursing versus non-nursing background of case managers**


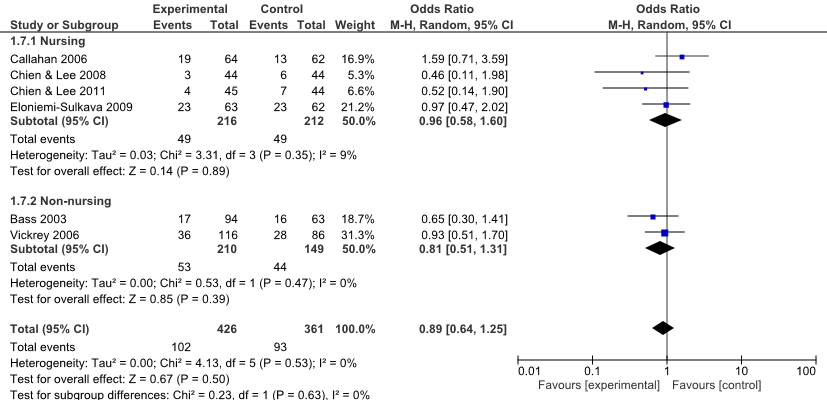


**Comparison 3: High contact versus low contact frequency**
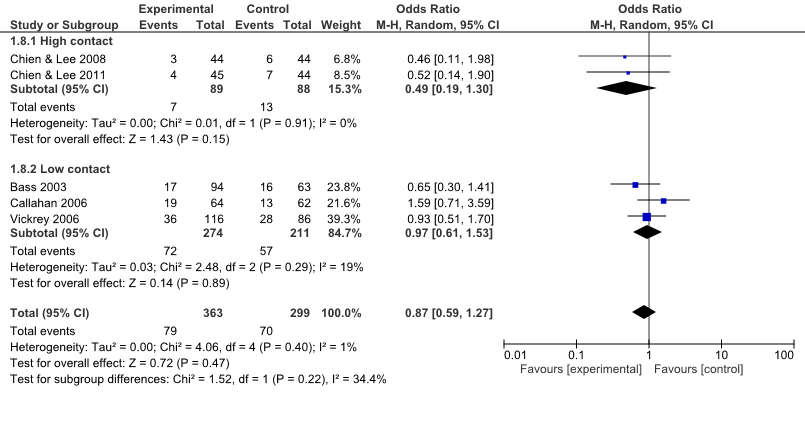


**Comparison 4: Supervision versus no supervision**


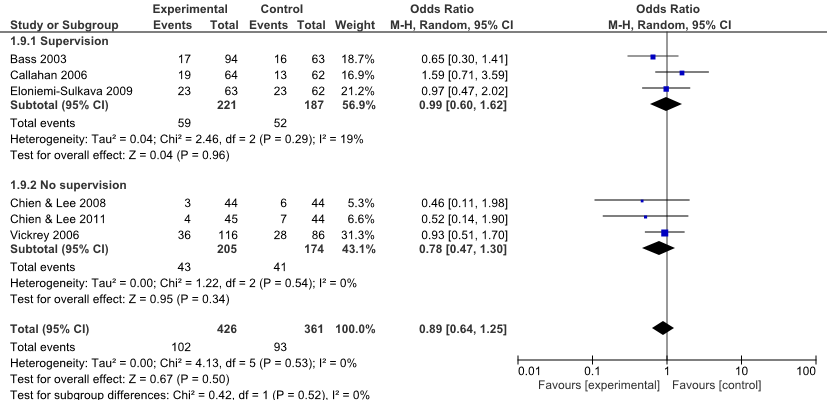


**Comparison 5: Low workload versus high workload**


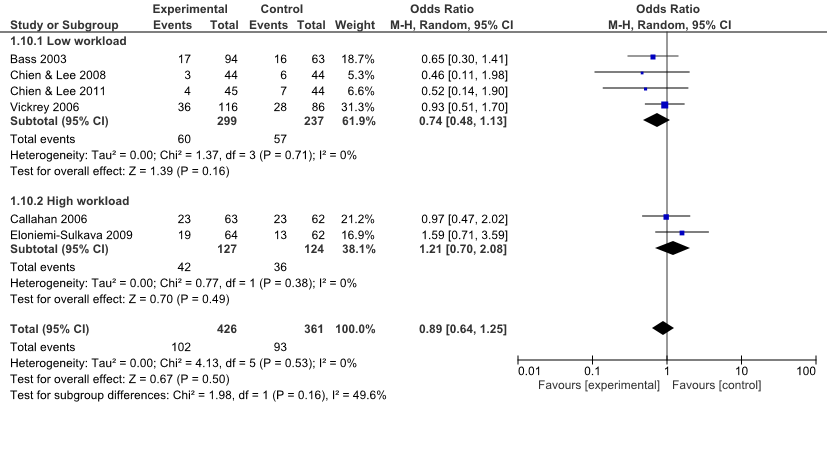


**Outcome 2: Institutionalisation**

**Comparison 1: Community base versus non-community base case managers**


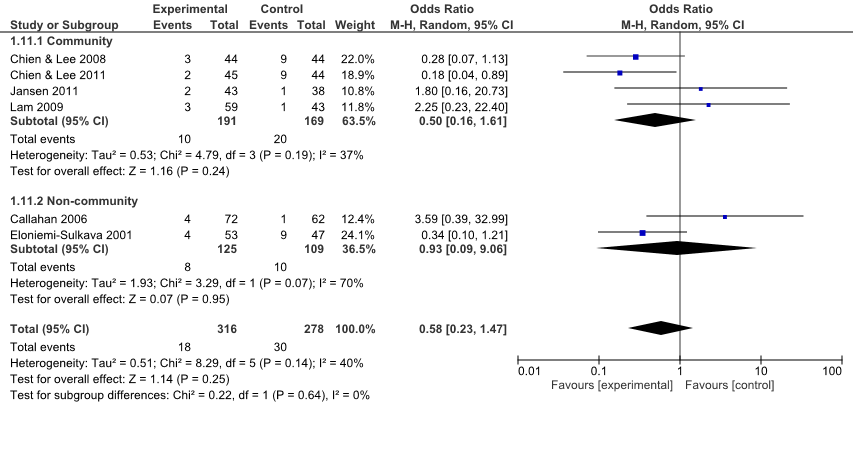


**Comparison 2: Nursing versus non-nursing background of case managers**


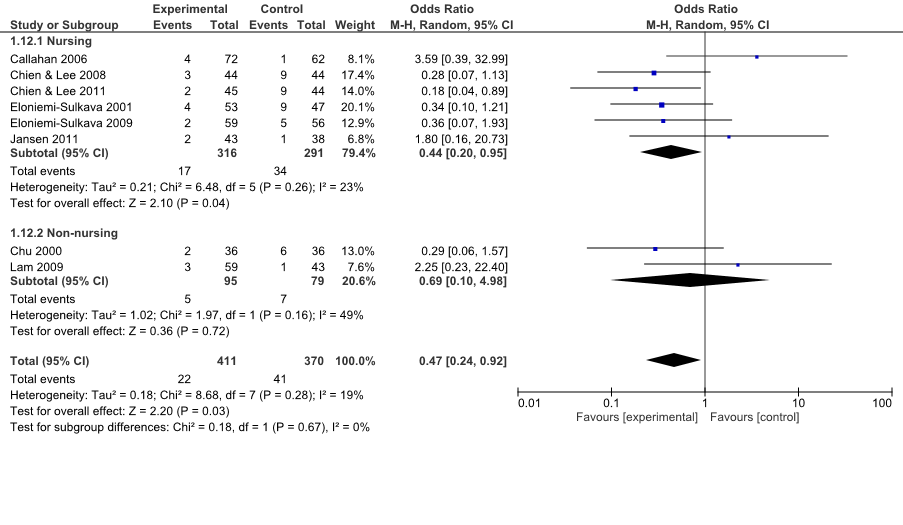


**Comparison 3: High contact versus low contact frequency**


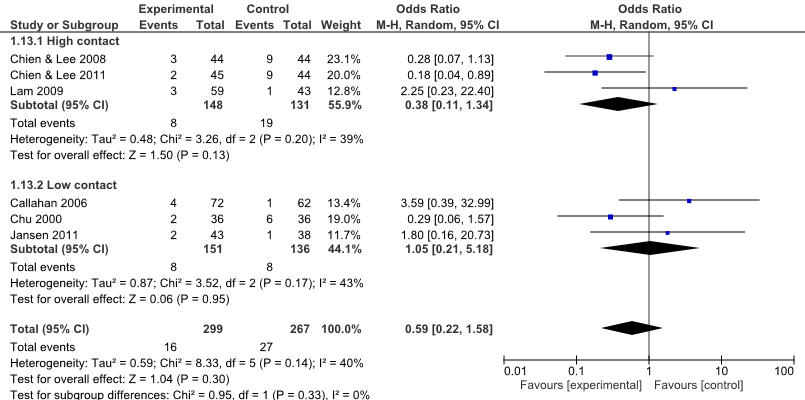


**Comparison 4: Supervision versus no supervision**


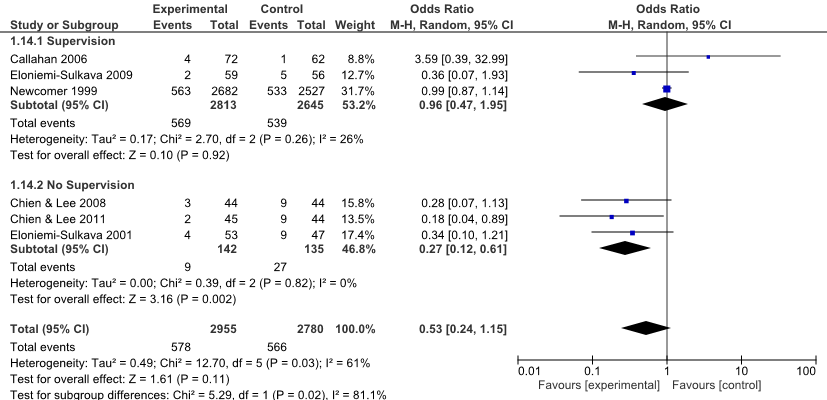


**Comparison 5: Low workload versus high workload**


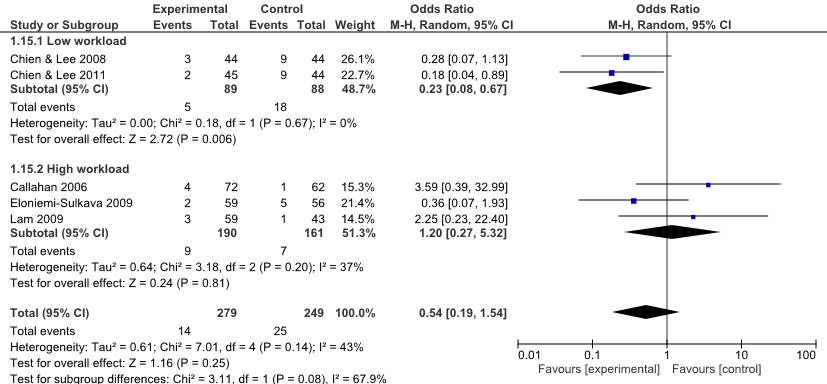


**Outcome 3: Mortality**

**Comparison 1: Community base versus non-community base case managers**


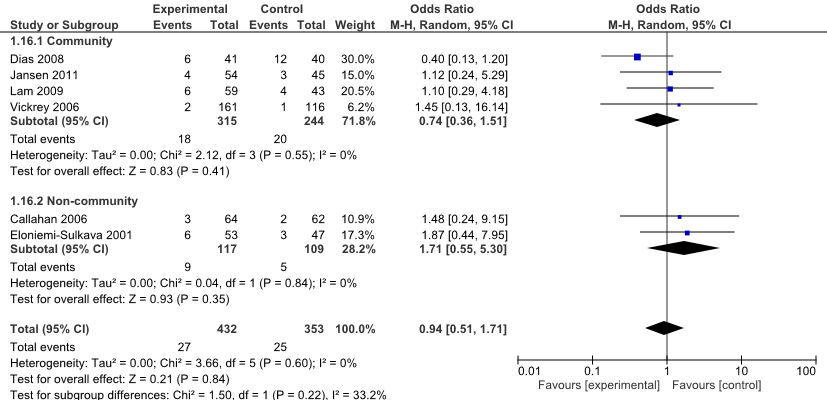


**Comparison 2: Nursing versus non-nursing background of case managers**


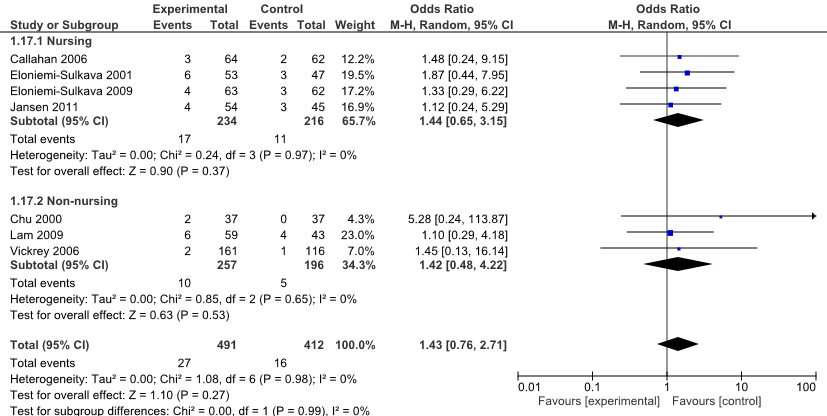


**Comparison 3: High contact versus low contact frequency**


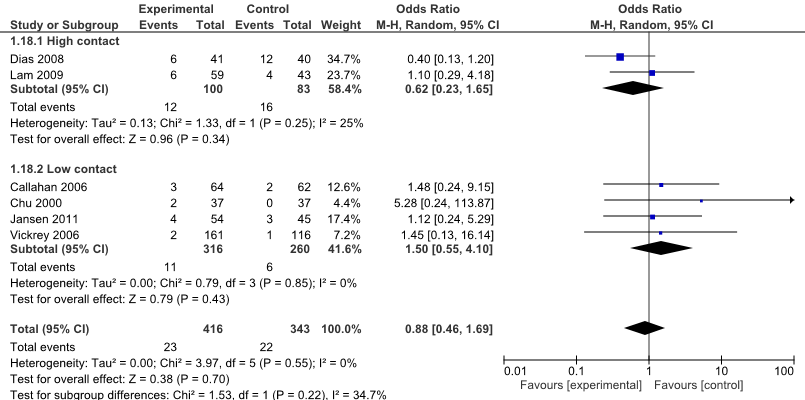


**Comparison 4: Low workload versus high workload**


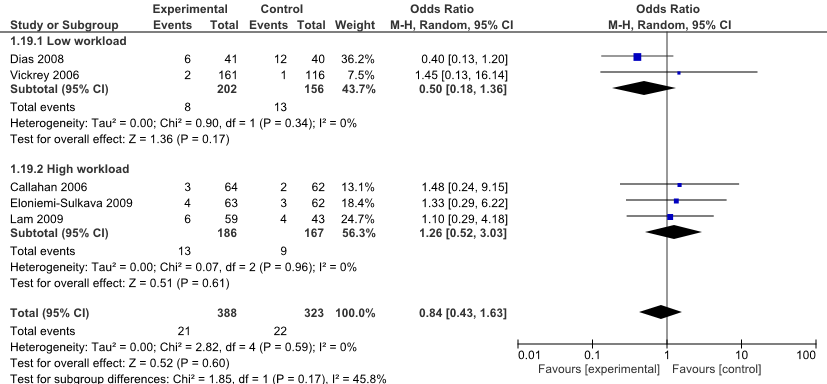


**Outcome 4: Patient cognition**

**Comparison 1: Community base versus non-community base case managers**


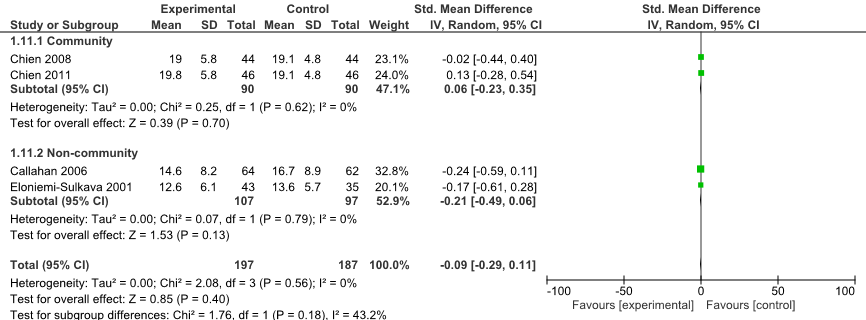


**Outcome 5: Caregiver burden**

**Comparison 1: Supervision versus no supervision**


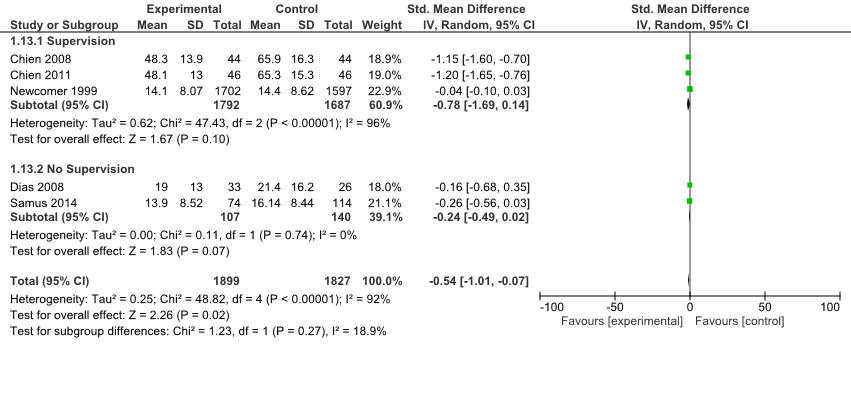


**Outcome 6: Caregiver mood**

**Comparison 1: High contact versus low contact frequency**


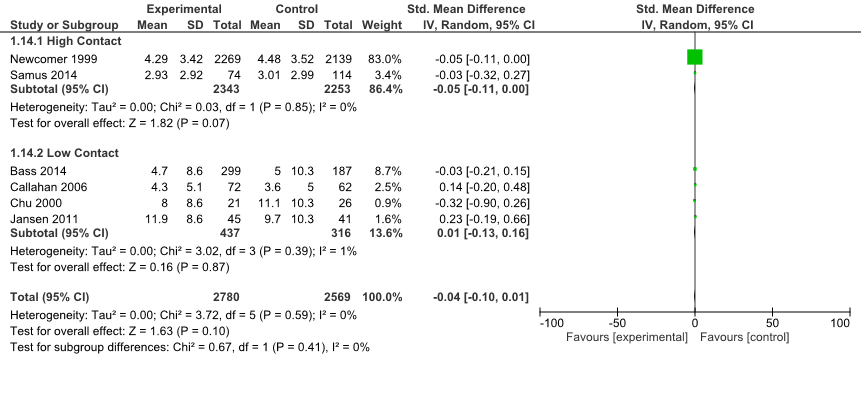


**Outcome 7: Caregiver quality of life**

**Comparison 1: Nursing versus non-nursing background of case managers**


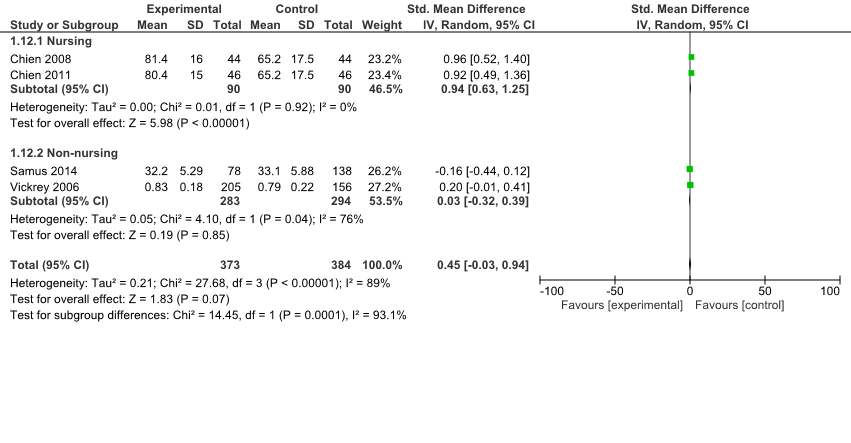


**Outcome 8: Patient behaviour**

**Comparison 1: Low workload versus high workload**


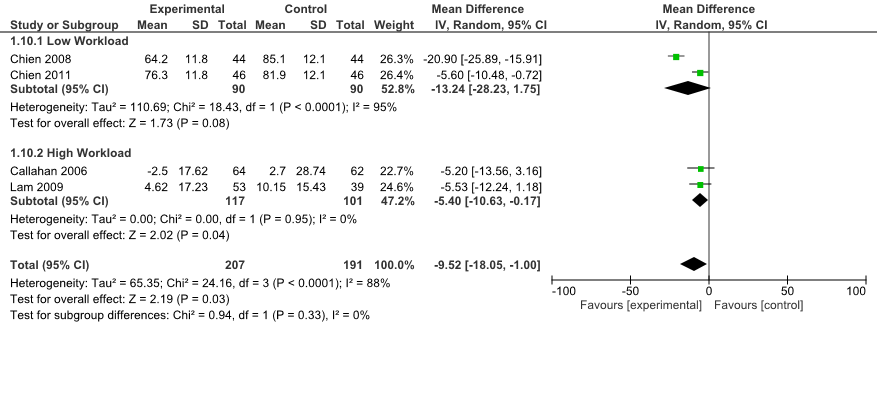

Supplement: Supplementary file 5 — Forest plots – all of the forest plots for the meta-analyses and the subgroup analyses. (DOCX 1725 kb) [file 12913_2017_2677_MOESM5_ESM.docx]
