# Supplementary material for: The effectiveness of community-based coordinating interventions in dementia care: a meta-analysis and subgroup analysis of intervention components
Source: BMC Health Serv Res. 2017 Nov 13;17:717. doi: 10.1186/s12913-017-2677-2 (PMC5683245; doi:10.1186/s12913-017-2677-2)

**Appendix 6: Funnel plots used to check publication bias**

**Outcome 1: Hospitalisation**


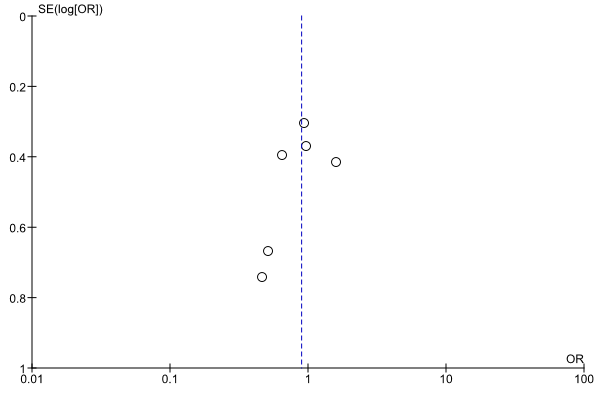


**Outcome 2: Institutionalisation**


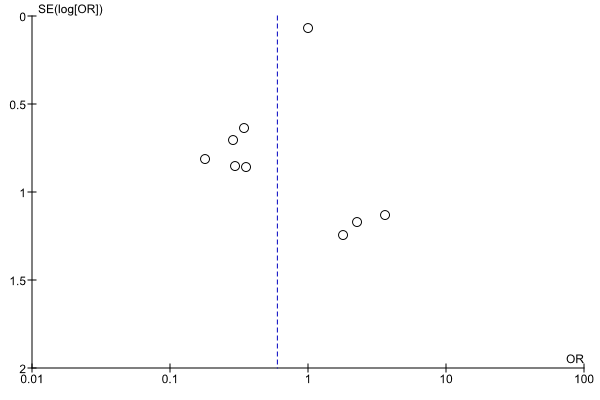


**Outcome 3: Mortality**


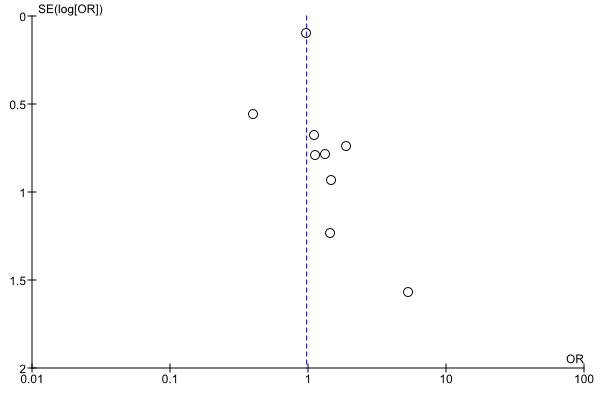


**Outcome 4: Patient quality of life**


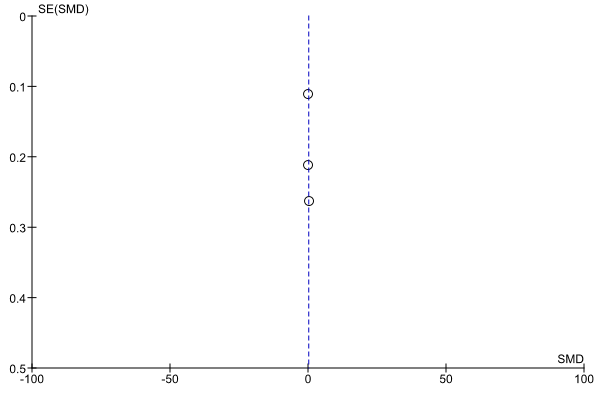


**Outcome 5: Patient cognition**


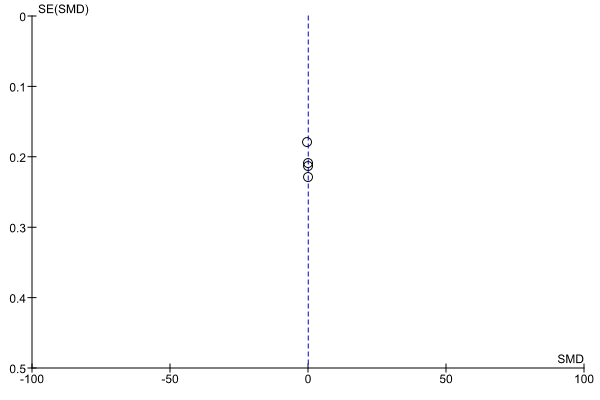


**Outcome 6: Patient function**


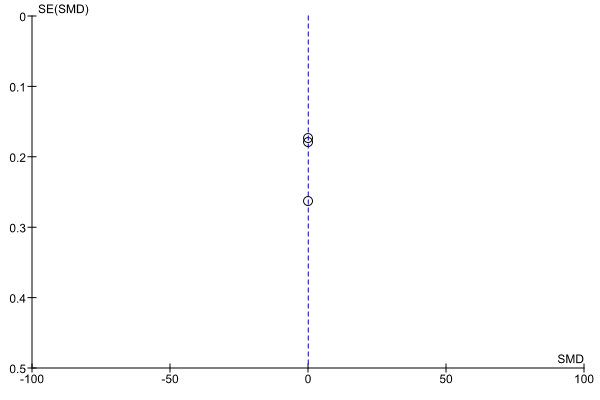


**Outcome 7: Caregiver burden**


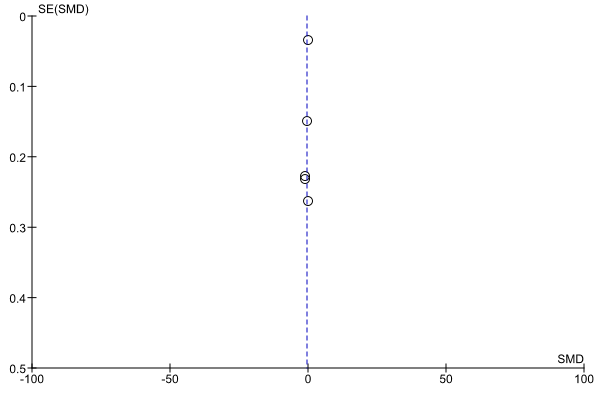


**Outcome 8: Caregiver mood**


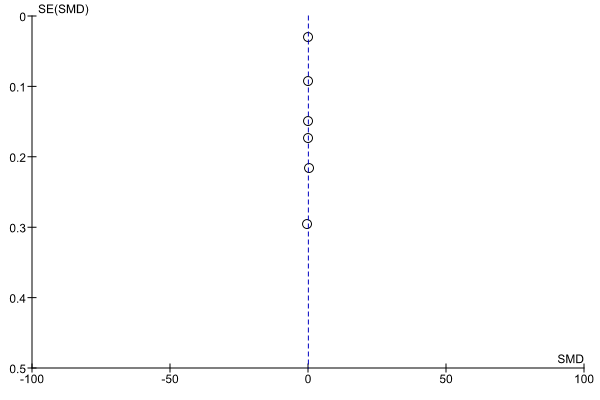


**Outcome 9: Caregiver quality of life**


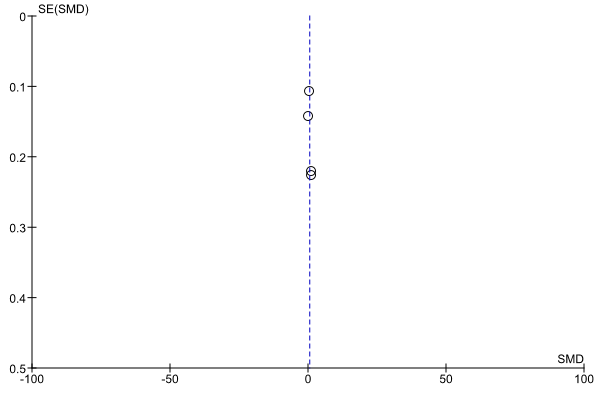


**Outcome 10: Social support**


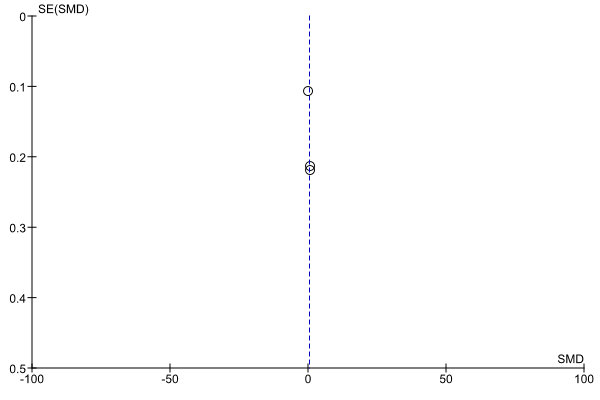


**Outcome 11: Patient behaviour**


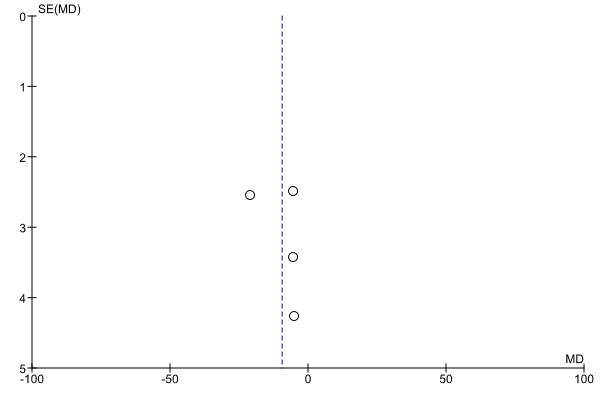


**Outcome 12: Patient depression**


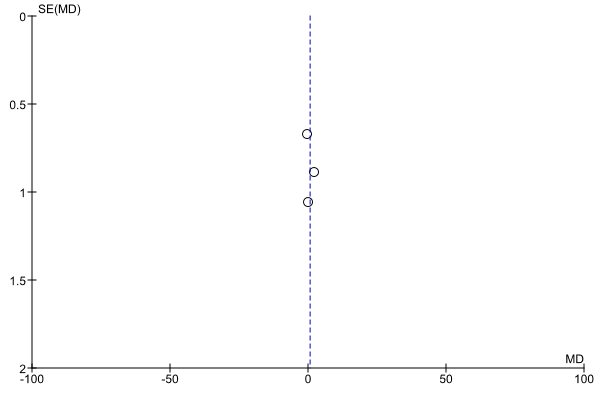

Supplement: Supplementary file 6 — Publication bias – all of the funnel plots to assess publication bias for each outcome measure. (DOCX 144 kb) [file 12913_2017_2677_MOESM6_ESM.docx]
